# Managing an Online Survey about Influenza Vaccination in Primary Healthcare Workers 

**DOI:** 10.3390/ijerph120100541

**Published:** 2015-01-09

**Authors:** Diana Toledo, Nicole Aerny, Núria Soldevila, Maretva Baricot, Pere Godoy, Jesús Castilla, Susana García-Gutierrez, Núria Torner, Jenaro Astray, José María Mayoral, Sonia Tamames, Fernando González-Candelas, Vicente Martín, José Díaz, Angela Domíguez, the CIBERESP Working Group for the Survey on Influenza Vaccination in Primary Health Care Workers

**Affiliations:** 1 CIBER Epidemiología y Salud pública (CIBERESP), Barcelona 08036, Spain; E-Mails: nuriasolde@gmail.com (N.S.); maretva@gmail.com (M.B.); pere.godoy@gencat.cat (P.G.); jesus.castilla.catalan@cfnavarra.es (J.C.); nuria.torner@gencat.cat (N.T.); fernando.gonzalez@uv.es (F.G.-C.); angela.dominguez@ub.edu (A.D.); 2 Departament de Salut Pública, Universitat de Barcelona, Barcelona 08036, Spain; 3 Subdirección de Promoción de la Salud y Prevención, Consejería de Sanidad, Madrid 28035, Spain; E-Mail: naerny@gmail.com; 4 Agencia de Salut Pública de Catalunya, Barcelona 08005, Spain; 5 Instituto de Salud Pública de Navarra, Pamplona 31003, Spain; 6 Unidad de Investigación, Hospital Galdakao-Unansolo (Osakidetza), Bizkaia 48960, Spain; E-Mail: susana.garciagutierrez@osakidetza.net; 7 Red de Investigación en Servicios de Salud en Enfermedades Crónicas (REDISSEC), Galdakao, Bizkaia 48960, Spain; 8 Área de Epidemiología, Comunidad de Madrid, Madrid 28035, Spain; E-Mail: jenaro.astray@salud.madrid.org; 9 Servicio de Vigilancia de Andalucía, Sevilla 41071, Spain; E-Mail: josem.mayoral.sspa@juntadeandalucia.es; 10 Dirección General de Salud Pública, Investigación, Desarrollo e Innovación, Junta de Castillay León, Valladolid 47071, Spain; E-Mail: TamGomSo@jcyl.es; 11 Unidad Mixta Genómica y Salud FISOBIO-Salud Pública, Universidad de Valencia, Valencia 46014, Spain; 12 Área de Medicina Preventiva y Salud Pública, Universidad de León, León 24071, Spain; E-Mail: vicente.martin@unileon.es; 13 Servicio Andaluz de Salud Pública, Sevilla 41071, Spain; E-Mail: jose.diazborrego.sspa@juntadeandalucia.es

**Keywords:** online survey, primary healthcare workers, research design, data collection, methodological considerations.

## Abstract

Online surveys are increasingly used due to their speed and efficiency. The aim of this study was to analyze factors that may have contributed to the quality and speed of response of an online survey on influenza vaccination in primary healthcare workers. A multicenter study including family physicians, nurses and pediatricians from primary healthcare teams from seven Spanish Autonomous Communities was designed. The centers were selected by simple random sampling. The survey remained active and accessible for 56 days and four reminders were sent. The odds ratios (OR) and their 95% confidence intervals (CI) were calculated to assess the association of sociodemographic variables and responding to the survey before the second reminder. Complete, validated information was obtained from 1965 primary healthcare workers. The total response rate was 36.2%. More nurses (46.3%) responded before the second reminder and more family physicians (52.8%) after the second reminder. The adjusted OR shows that family physicians responded later (AOR 1.46, 95% CI 1.2–1.8) than nurses. The responses obtained in the first 24 h after the initial sending and the reminders accounted for 41.7% of the completed surveys, indicating the importance of reminders.

## 1. Introduction

The use of online surveys as research tools became widespread in the second half of the 1990s. Currently, there are tools that simplify the sending of these surveys, allow reminders to be made and automatically tabulate the results [1]. Some studies have shown the advantages and limitations of online surveys compared with traditional surveys (telephone, post or personal interview) [2,3,4,5]. Online surveys may be used in different fields and target different populations. In recent years, the use of online surveys in research studies aimed at healthcare workers has increased [3,4].

The main advantages of online surveys are that they collect information in real time, have a low cost, facilitate contact with scattered groups, make results easier to analyze, may eliminate interviewer bias and may be carried out more rapidly than other techniques [3,5,6].

There are three main types of drawback: online surveys usually achieve a lower response rate; there may be doubts about their reliability and validity; and, participants may be reluctant to participate due to confidentiality concerns [2,3,7]. For these reasons it is important that the quality of the design and the communication strategy are carefully considered.

The sources of error in surveys are an increasingly-important research topic in the social sciences. Currently, the theoretical framework of total survey error is used to identify any errors that may arise in the design, collection, processing and analysis of data [6,8]. Total survey error classifies errors in two categories: sampling errors and non-sampling errors. Sampling errors may occur due to the choice of the type of sample, its size or the chosen estimators. Non-sampling errors include errors of coverage, measurement, nonresponse, specification and processing [6,8,9,10,11,12].

Coverage error occurs when part of the target population of the study is not included. Measurement error occurs when an answer is incorrect due to reasons related to the respondent, the interviewer or the design of the questionnaire, although there is evidence that, in online surveys, the bias associated with social desirability (the tendency of a person to provide an answer they believe is socially more acceptable) is less frequent than in other types of questionnaires [13]. Non-response error occurs when the person the survey is aimed at does not respond or does not answer all questions. Specification error occurs when the concept involved in the survey question is not the same as that the question was intended to measure. Finally, processing error refers to any errors that may arise during the processing of survey data.

Given that influenza vaccination coverage in the population considered at risk of influenza complications in Spain (59.1% aged ≥ 65 years) [14] was well below the 75.0% recommended by the WHO and the European Union [15,16], we believed that investigation of healthcare workers’ knowledge of and attitudes to influenza vaccination was warranted, as this group plays an important role in the vaccination of the patients they serve [17,18,19,20]. A study to investigate the association between influenza vaccination of primary healthcare workers and knowledge of and attitudes towards influenza vaccination and disease in Spain was carried out [21]. The aim of this study was to analyze the factors contributing to increase the response rate and quality of the online survey on the knowledge of and attitudes to influenza vaccination of primary healthcare professionals (doctors, pediatricians and nurses).

## 2. Materials and Methods

### 2.1. Sampling Procedure

All primary healthcare workers potentially involved in influenza vaccination (family physicians, pediatricians and nurses) from seven Autonomous Communities were considered eligible to participate. In Spain there are 17 Autonomous Communities, but only the largest ones (Andalusia, Castile & Leon, Catalonia, Valencia, Madrid, Navarra and the Basque Country) which represent 69.9% of the Spanish population, were included in the study.

The sample size necessary to achieve the study objectives was estimated considering a bilateral alpha error of 0.05, a statistical power of 0.8, a prevalence rate of the factors considered of 0.7 and an odds ratio of the prevalence of these behaviors in vaccinated compared with non-vaccinated subjects of 2.0. Therefore, the minimum number of primary healthcare workers required for inclusion was estimated at 185. In addition, as we proposed to analyze the study objectives according to different strata (age group, type of profession and years of practice), the minimum number of participants required was estimated as 925. Taking into account reports that show the proportion of responses obtained by the administration of online surveys is about 30.0% [22,23], the minimum number of surveys required to be sent was 3,083. Each participant Autonomous Community provided a list of 30 primary care centers selected by simple random sampling.

### 2.2. Survey Instrument and Pilot Testing

The study was conducted using an online survey distributed through a list of institutional e-mail addresses. The questionnaire was designed based on the review and compilation of instruments used in scientific publications related to the study topic, mainly the questionnaire used by Kraut *et al*. [22]. Questions were adapted to the specific circumstances of the Spanish National Health System, ensuring conceptual and semantic equivalence.

The final questionnaire consisted of 23 questions (22 closed and one open). Questions were distributed in three sections: information on the history of chronic disease and vaccination, knowledge of influenza and the influenza vaccine and sociodemographic information.

The questionnaire was tested on three occasions in a group of 20 healthcare workers to assess readability. On the first two occasions, the questionnaire was tested on paper in order to identify questions whose wording might be confusing and to study the time required to respond. The average response time was 10 min. The suggested modifications were made on the online version. The survey was designed and information collected using the web platform: www.encuestafacil.com.

The questions were structured to appear gradually, spread over a total of six screens. The first screen welcomed the participants and provided general information on the survey. The following four screens contained the survey questions, and the last screen contained a text thanking the participants and a link to the website of one of the participating entities. Using the tools provided by the web platform, 19 of the 23 questions were compulsory, *i.e.*, they had to be answered in order to access the following question. Once the design of the questionnaire on the web platform was completed, the third test was used to ensure understanding of the wording, that the survey was fluid, and that the time required for the survey to be completed online was similar to that estimated previously.

### 2.3. Study Procedure

A coordinating team, made up by one representative from each participating Autonomous Community, was set up to ensure the follow up of the study [24]. Selected primary healthcare workers were contacted through the list of institutional e-mail addresses by sending an invitational e-mail containing the presentation of the study and the link to access the survey.

The web platform allowed lists of the e-mails for each participating community to be drawn up. As the e-mail addresses were loaded, the platform validated and identified any that could cause problems, thus permitting corrections to be made. Each e-mail address was assigned a code number.

The survey was active and accessible for 56 days in each Autonomous Community between 1 and 25 May 2012. The study was planned to send reminders every ten days during the study period. The reminders were sent early on Monday morning on days 12, 22, 33 and 43 after the initial e-mail.

As the computers in the primary healthcare centers may have been used by more than one employee, the web platform was configured to ensure that each e-mail address had a specific link associated with it. Therefore, if a questionnaire was left half completed, when the participant accessed the questionnaire again, they were taken to the place where they had left it.

One feature of the web platform was that it allowed each participant to save the responses completed and continue later, to go forward or go back to the different screens, to abandon the questionnaire, or to finalize the questionnaire once all questions had been answered. This allowed the e-mail addresses of primary healthcare workers who had completed the questionnaire or had begun and then expressly abandoned it to be excluded from the list for the sending of reminders.

We offered participating centers the possibility of receiving a participation certificate and the option of receiving a personal certificate for each individual participant which had to be requested by e-mail.

### 2.4. Statistical Analysis

The data analysis included all the primary healthcare workers who completed the survey. Partially-answered surveys were eliminated from the final analysis.

A bivariate comparison using the Chi-square test was made between all primary healthcare workers invited to the survey and those who completed the survey considering the sociodemographic variables.

The odds ratios (OR) and their 95% confidence intervals (CI) were calculated to assess the association of sociodemographic variables and responding to the survey before the second reminder. In order to control for possible confounding variables, the adjusted OR was calculated using logistic regression, including sex and professional category in the model. A two-sided *p*-value < 0.05 was considered to indicate a statistically significant difference. The analysis was performed using SPSS version 18 (SPSS Inc., Chicago, IL, USA).

### 2.5. Ethical Issues

All information collected was treated as confidential in strict compliance with the norms pertaining to observational studies. Participants gave consent by accessing the link provided in the e-mail inviting them to participate in the study. The invitational e-mail containing the presentation of the study stated that all responses would be anonymized. In the stored data, respondents were identified only by a number. The study protocol, including the consent procedure was approved by the Clinical Research Ethics Committee of the Jordi Gol Institute for Research in Primary Healthcare.

## 3. Results and Discussion

### 3.1. Results

**Figure 1:**
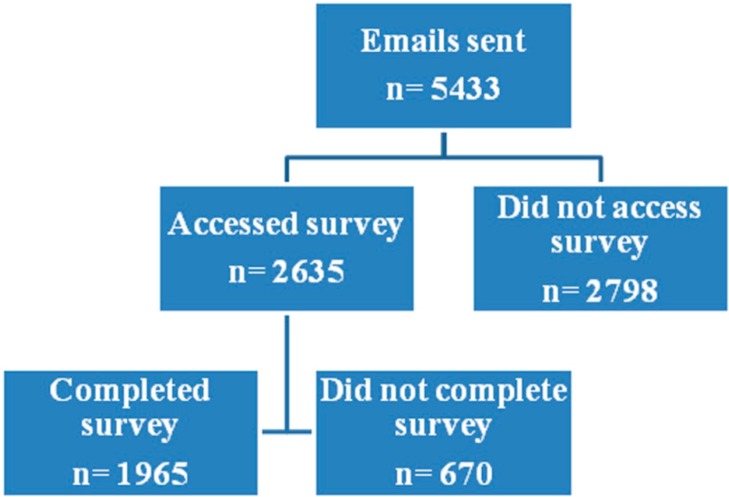
Dynamics of the Survey.

The survey was sent to 5,433 primary healthcare workers; 3291 opened the e-mail invitation, of these 2635 (48.5%) accessed the survey and responded fully or partially and 1965 (36.2%) completed the survey (Figure 1). Just 32 certification requests were received.

#### 3.1.1. Characteristics of participants

Of the 1965 primary healthcare workers who completed the survey, 42.6% were aged 45–54 years, 74.4% were female, 47.0% were family physicians and 65.4% had 10–29 years professional experience (Table 1).

**Table 1:** Sociodemographic characteristics of primary healthcare workers who completed the survey.

Variable	Categories	Completed Surveys (*n* = 1965)
Age group (in years)	25–34	137 (7.0%)
	35–44	493 (25.1%)
	45–54	837 (42.6%)
	≥55	498 (25.3%)
Sex	Male	503 (25.6%)
	Female	1462 (74.4%)
Professional category	Family physician	923 (47.0%)
	Pediatrician	203 (10.3%)
	Nurse	839 (42.7%)
Years in profession	<10	159 (8.1%)
	10–29	1286 (65.4%)
	≥30	520 (26.5%)

Table A1 shows basic sociodemographic information (age, sex and professional category) of all the primary healthcare workers who received the invitation to the survey and those who completed the survey. Those belonging to the 35–44 years age group were the most frequent participants in relation to all the primary healthcare workers invited to the survey (25.1% and 20.5%, respectively; *p* ≤ 001) and those older than ≥55 years age group were the ones who participated less (25.3% and 30.7%, respectively; *p* ≤ 0.001). The participation rate was higher in women than in men (74.4% and 71.8%, respectively; *p* ≤ 0.01) and in nurses than in medical doctors (42.7% and 37.8%, respectively; *p* ≤ 0.001).

#### 3.1.2. Evolution of Response Rate

**Figure 2:**
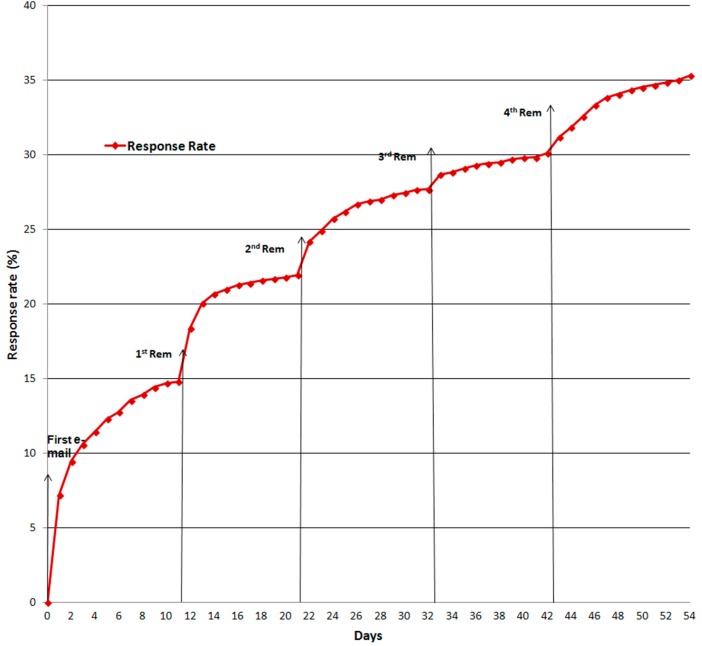
Evolution of the response rate after initial e-mail and reminder e-mails.

The cooperation rate (proportion of primary healthcare workers who accessed the survey) was 48.5% and the response rate (proportion of primary healthcare workers who completed the survey) was 36.2%. Figure 2 shows the evolution of the response rate during the period the survey was accessible and the effect of the reminders in increasing the response rate in the 24 h after sending.

The response rate within 24 h after the initial e-mail was 7.2%. In the following days, the increase in the daily response rate fell until the first reminder was sent, when the response rate increased from 14.8% to 18.4% in the first 24 h. In the following days, the response rate fell again. After the second reminder, the response rate increased from 22.0% to 24.2% in the first 24 h. The same pattern was observed for the third and fourth reminders, although the increase in the response rate in the first 24 h was only 1.0% each time (27.7% to 28.7% and 30.1% to 31.2%, respectively).

Of the 1965 primary healthcare workers who completed the survey, 60.8% responded before the second reminder was sent (during the first twenty-one days) and 39.2% after it was sent (Table 2). In both groups, the most frequent age group was 45–54 years (42.8% and 42.3%, respectively), females (76.5% and 71.2%, respectively) and primary healthcare workers with 10–29 years of professional experience (65.9% and 64.7%; respectively). More nurses (46.3%) responded before the second reminder and more family physicians (52.8%) after the second reminder. The adjusted OR shows that family physicians responded later than nurses (aOR 1.46, 95% CI 1.2–1.8).

**Table 2:** Distributionofresponses before and after sending the second reminder.

Variable	Categories	Response before the Second Reminder (*n* = 1194)	Response after the Second Reminder (*n* = 771)	Crude OR (95% CI)	*p* value	Adjusted OR ^a^ (95% CI)	*p* value
Age	25–34	85 (7.1%)	52 (6.7%)	1		1	
	35–44	317 (26.5%)	176 (22.8%)	0.91 (0.6–1.3)	0.63	0.82 (0.6–1.2) ^a^	0.32
	45–54	511 (42.8%)	326 (42.3%)	1.04 (0.7–1.5)	0.83	0.93 (0.6–1.4) ^a^	0.71
	≥55	281 (23.5%)	217 (28.1%)	1.26 (0.9–1.9)	0.24	1.11 (0.8–1.7) ^a^	0.61
Sex	Female	913 (76.5%)	549 (71.2%)	1		1	
	Male	281 (23.5%)	222 (28.8%)	1.31 (1.1–1.6)	0.009	1.14 (0.9–1.4) ^b^	0.25
Professional category	Nurse	553 (46.3%)	286 (37.1%)	1		1	
	Family physician	516 (43.2%)	407 (52.8%)	1.53 (1.3–1.9)	<.001	1.46 (1.2–1.8) ^c^	<.001
	Pediatrician	125 (10.5%)	78 (10.1%)	1.21 (0.9–1.7)	0.25	1.17 (0.9–1.6) ^c^	0.33
Years in profession	<10 years	96 (8.0%)	63 (8.2%)	1		1	
	10–29 years	787 (65.9%)	499 (64.7%)	0.97 (0.7–1.4)	0.84	0.93 (0.7–1.3) ^a^	0.67
	>30 years	311 (26.0%)	209 (27.1%)	1.02 (0.7–1.5)	0.90	1.03 (0.7–1.5) ^a^	0.30

^a^ Adjusted by sex and professional category; ^b^ Adjusted by professional category; ^c^ Adjusted by sex.

### 3.2. Discussion

The design and implementation of an online survey as a tool to investigate the attitudes to and knowledge of influenza vaccination in primary healthcare workers was satisfactory. The use of the theoretical framework of the total survey error and correct application of the methodology in the design permitted control of the appearance of possible sampling and non-sampling errors.

The overall response rate to the survey was 36.2%, similar to the mean response rates reported by other studies using a similar study population and online surveys [22,23,25,26,27,28,29].

The sample design allowed the opinions and attitudes of the population studied to be determined. The pilot survey consisted of several steps that ensured the quality of the questionnaire, although it was not possible to measure possible errors due to responses conditioned by social desirability [13].

A total of 12.3% (670) of the primary healthcare workers invited to participate did not complete the questionnaire or returned it uncompleted [29], a percentage similar to other studies, which also did not identify reasons for this behavior [30,31]. Our results show that family physicians responded later than other primary healthcare workers. Possible explanations could be the time taken to complete the survey or a heavy workload in primary care centers, although the reasons for non-completion were not sought.

Using personal interviews Picazo *et al.* have obtained a participation of 53.0% in primary healthcare workers and 69.0% in hospital specialists [32]. Using a paper surveys La Vela *et al.* in spinal cord injury centers obtained a response rate of 66.0% in healthcare workers working in an inpatients units and only 7.0% healthcare workers working in outpatient units [33].

Non-response bias is increasingly common in surveys and study of the reasons for this could identify less-well-represented groups. Our results show primary healthcare workers aged ≥55 years responded less than other age groups. It may be suggested that primary healthcare workers aged ≥55 years may have more limited knowledge or experience of the Internet [34].

The response obtained in the 24 h after the initial e-mail and the four reminders accounted for 42.0% of the completed surveys, but was only 7.2% after the first e-mail, reinforcing the importance of using reminders in this type of surveys.

Sending four reminders during the study increased the response rate and further underlined the importance of sending reminders in studies using online surveys [27,30,34,35,36]. There is a consensus that the first reminder should be sent between days 8 and 12 [23,28,34,35]. However, there is no consensus on the total number of reminders. Some authors [22] consider that one reminder is sufficient while others [26,28,29,30,34] suggest two or four reminders should be sent. Other studies coincide with our results in showing that the greatest increase in the response rate was observed after the first reminder [23,35,36]. In our study, the response rate fell gradually after reminders two, three and four were sent, although, together, they contributed to an appreciable increase in the response rate.

An online survey of United States healthcare workers by Pace *et al.* [37] and a similar survey by Parisi *et al.* [38] in healthcare workers from four primary healthcare areas in the USA used economic incentives as a way to increase the response rate. Our study offered participants the possibility to obtain a participation certificate and obtained a response rate similar to or higher than these authors. The use of institutional e-mail addresses as the main route of contacting primary healthcare workers was a strength of the study as it ensured that the survey reached the target population.

Using the online survey allowed information to be analyzed in real time and facilitated the production of reports for the participating Autonomous Communities and the detection of anomalies during the study period.

A limitation of the study was that the lists of e-mail addresses of primary healthcare workers were not up to date, with some addresses having been changed and other professionals no longer active. Although these problems were resolved, we do not know whether this may have influenced non-responses.

## 4. Conclusions

The choice of an online survey was beneficial and appropriate. The responses obtained in the first 24 h after the initial e-mail was 7.2%, which increased to 42% in the 24 h after reminders were sent. Our results highlight the importance of a good communication strategy for sending reminders, especially in the first stage of the implementation of a survey.

## Appendix

**Table A1:** Distribution of characteristics of all healthcare workers the survey was sent to and those finally analyzed.

Variable	Categories	All Healthcare Workers the Survey Was Sent (*n* = 5433)	Healthcare Workers Analyzed (*n* = 1965)	*P* Value
		*n* (%)	*n* (%)	
Age	<24	5 (0.1%)	--	--
	25–34	326 (6%)	137 (7%)	--
	35–44	1114 (20.5%)	493 (25.1%)	0.65
	45–54	2320 (42.7%)	837 (42.6%)	0.16
	≥55	1668 (30.7%)	498 (25.3%)	0.003
Sex	Male	1576 (29%)	503 (25.6%)	--
	Female	3857 (71%)	1462 (74.4%)	0.004
Professional category	Family physician	2535 (46.7%)	923 (47%)	--
	Pediatrician	591 (10.9%)	203 (10.3%)	0.52
	Nurse	2307 (42.5%)	839 (42.7%)	0.98
